# Citrinin Determination in Food and Food Supplements by LC-MS/MS: Development and Use of Reference Materials in an International Collaborative Study

**DOI:** 10.3390/toxins13040245

**Published:** 2021-03-30

**Authors:** Emmanuel K. Tangni, François Van Hove, Bart Huybrechts, Julien Masquelier, Karine Vandermeiren, Els Van Hoeck

**Affiliations:** 1 Sciensano, Chemical and Physical Health Risks, Organic Contaminants and Additives, Leuvensesteenweg 17, 3080 Tervuren, Belgium; Bart.Huybrechts@sciensano.be (B.H.); Julien.Masquelier@sciensano.be (J.M.); Karine.Vandermeiren@sciensano.be (K.V.); Els.VanHoeck@sciensano.be (E.V.H.); 2 Earth and Life Institute-Applied Microbiology (ELIM), Université catholique de Louvain (UCL), Croix du Sud 2 bte L7.05.06, 1348 Louvain-la-Neuve, Belgium; f.vanhove@yahoo.fr

**Keywords:** citrinin, reference materials, food, food supplements, red yeast rice, *Ginkgo biloba*, LC-MS/MS, method validation study

## Abstract

The development of incurred reference materials containing citrinin (CIT) and their successful application in a method validation study (MVS) in order to harmonize CIT determination in food and food supplements are demonstrated. CIT-contaminated materials made of red yeast rice (RYR), wheat flour, and *Ginkgo biloba* leaves (GBL), as well as food supplements made of red yeast rice (FS-RYR) and *Ginkgo biloba* leaves (FS-GBL), were manufactured in-house via fungal cultivation on collected raw materials. The homogeneity and stability from randomly selected containers were verified according to the ISO 13528. CIT was found to be homogenously distributed and stable in all contaminated materials, with no significant degradation during the timescale of the MVS when storage was performed up to +4 °C. Next, an MVS was organized with eighteen international laboratories using the provided standard operating procedure and 12 test materials, including three RYRs (blank, <50 µg/kg, <2000 µg/kg), two wheat flours (blank, <50 µg/kg), two GBL powders (blank, <50 µg/kg), three FS-RYRs (blank, <50 µg/kg, <2000 µg/kg), and two FS-GBLs (blank, <50 µg/kg). The results of seven CIT-incurred materials showed acceptable within-laboratory precision (RSDr) varying from 6.4% to 14.6% and between-laboratory precision (RSDR) varying from 10.2% to 37.3%. Evidenced by HorRat values < 2.0, the results of the collaborative trial demonstrated that the applied analytical method could be standardized. Furthermore, the appropriateness of producing CIT reference materials is an important step towards food and feed quality control systems and the organization of proficiency tests.

## 1. Introduction

Mainly produced by *Aspergillus*, *Penicillium*, and *Monascus* fungi, citrinin (CIT) is a mycotoxin that occurs often in stored grains, fruits, vegetable juices, herbs, and spices [1,2]. CIT is nephrotoxic, genotoxic, and carcinogenic [3,4]. In 2012, the EFSA assessed the health risk related to the presence of CIT in food, and concluded that more data regarding the occurrence of CIT in food are needed in order to enable refinement of the risk assessment [1]. In addition, the use of red yeast rice (RYR) in dietary food supplements has gained popularity in Europe due to its ability to achieve and maintain healthy cholesterol levels [5]. RYR is prepared upon fermentation of rice grains polluted by *Monascus purpureus* that produce monacolins (i.e., inhibitors of cholesterol production). As a result, RYR could have a significant potential to reduce healthcare costs and contribute to public health by reducing heart disease risk in individuals with moderate elevations of circulating cholesterol levels [6,7]. Even though many patients use worldwide RYR as an alternative therapy for hyperlipidemia, the discovery of a toxic fermentation byproduct, CIT, causes much controversy about the safety of RYR [8]. Based on the risk assessment carried out by the EFSA, the European Regulation (EC) No 212/2014 [9] laid down a maximum limit (ML) of 2000 μg/kg for CIT in FS-RYR. Taking into account more recent data, this ML has been lowered to 100 µg/kg [10]. Nowadays, medicinal and aromatic herb consumption is also increasing due to its therapeutic or natural properties, which may concomitantly lead to an increase of mycotoxin intake. For example, *Ginkgo biloba* leaves (GBL) from Spain contained up to 355 µg of CIT/kg, together with 23.3 µg of aflatoxins/kg and 1.1 µg of ochratoxin A/kg [11]. In order to gather reliable occurrence information to support food safety policy, it is necessary to have adequate analytical techniques for the detection of CIT in food. Moreover, detection of mycotoxins can be achieved by various analytical methods, such as gas chromatography–mass spectrometry (GC-MS) and high-performance liquid chromatography (HPLC) in combination with fluorescence and/or UV detection or immunodiagnostic tools [12,13]. However, during the last 10 years, liquid chromatography–tandem mass spectrometry (LC-MS/MS) has become the universal technique for mycotoxin analysis, and it has been widely applied for various matrices [14,15]. Recently, occurrence data of CIT and its biomarkers were obtained in a handful of monitoring studies performed hitherto in Europe [16,17]. So far, CIT was not targeted among the 72 official methods published by the Association of Official Agricultural Chemists (AOAC), the European Committee for Standardization (CEN), and the International Organization for Standardization (ISO) for mycotoxin analysis in food [18]. Furthermore, Solfrizzo et al. [14] stated that there is a need for the harmonization of mycotoxin determination to ensure the verification of compliance with feed and food law and animal health and animal welfare rules. In this context and within the framework of the European Regulation (EC) 882/2004 on official controls [19], the EC issued the mandate M/520 pertaining to 11 standardization priorities of analytical methods for mycotoxin determination in food [20]. Of the 11 priorities of the mandate, the secretary of CEN/TC275 assigned via the working group 5 (WG5) one on the “determination of CIT in food by LC-MS/MS” to the Belgian National Reference Laboratory. The process for the standardization was undertaken via a method validation study (MVS) using the in-house prepared CIT matrix reference materials. Indeed, matrix reference materials play a key role for internal and external validation processes [21]. They are important tools for laboratories for method development and validation, quality control evaluation, and inter-laboratory comparisons [22]. The present paper describes the preparation of the incurred CIT reference materials and their use in the MVS in order to assess the inter-laboratory repeatability and reproducibility for CIT determination in food and food supplements, as requested in the standardization procedure.

## 2. Results and Discussion

### 2.1. Samples, Homogeneity, and Stability Assessment in Contaminated Materials

Five blank matrices (CIT < LOQ) and seven CIT contaminated samples were provided to each participant of the present method validation study. These 12 samples were coded from A to L (Table 1).

Homogeneity and stability were evaluated using a previously published LC-MS/MS method [15]. This method was successfully validated to meet the criteria laid down in EC decision 2002/657/EC [23] and EU 519/2014 [24] in FS-RYR and wheat, achieving a validated LOQ of 2.5 µg/kg. Note that the average between-day recoveries varied between 70–120% [24], while the expanded measurement uncertainties MU_(k=2)_ were 30% for FS-RYR and 40% for wheat. To verify the accuracy and reliability of this method, the laboratory participated in a proficiency test organized by FAPAS for maize flour, resulting in a Z-score of 1.0 for an assigned value of 87.9 µg/kg. Based on the accepted |Z-score| ≤ 2.0 criterion, it can be concluded that satisfactory results are achieved with this analytical method [25].

The tests for adequate and sufficient homogeneity were conducted according to the criteria of ISO-13528 [26] and IUPAC [27]. More details are given in Tangni et al. [28]. All of the materials proved to be adequately homogeneous (Table 2), and an acceptable degree of variability in the distributed units of each test material was guaranteed.

Next, the stability of the samples was evaluated at different temperatures (−20 °C, +4 °C, and 24 °C) for the period starting at the day that the samples were dispatched until the date for data submission (Table 3).

(1) $$\mathrm{Ratio}\;=\;|\mathrm{Level}(4\;{}^{\circ}C/24\;{}^{\circ}C)\;-\;\mathrm{Level}(-20\;{}^{\circ}C)|/\sqrt{MU_{\left(-20{}^{\circ}C\right)}{}^{2}+MU_{\left(+4{}^{\circ}C\;or+24{}^{\circ}C\right)}{}^{2}}\;({\mathrm{MU}}_{\left(k\;=\;2\right)}\;=\;2\;\times \;{\mathrm{SD}}_{\left(2\;\mathrm{averages}\right)}/\surd 2)$$

for each contaminated material stored at +4 °C and +24 °C compared to the samples stored at −20 °C. Ratio of changes in CIT contents (Equation 1) was significant if ratio > 1.

Most of the provided materials proved to be adequately stable at +4 °C for at least three months, despite some deviations observed for GBL and FS-RYR_low level_ upon three months of conservation (Table 3). Consequently, it was recommended that the materials of FS-GBL and FS-RYR be analyzed until three months after reception. The stability study indicated that storage of the materials at +24 °C should be avoided.

For the present study, it is noticeable that most of the participating laboratories (13) have stored the samples at −20°/−18 °C before performing the analyses, while five laboratories stored the test materials at +4 °C storage.

The analytical method was drafted as the standard operating procedure (SOP) in CEN format and distributed for validation to the participants with instructions.

### 2.2. Participants’ Locations and Experiences in Mycotoxin Analyses

**Figure 1:**
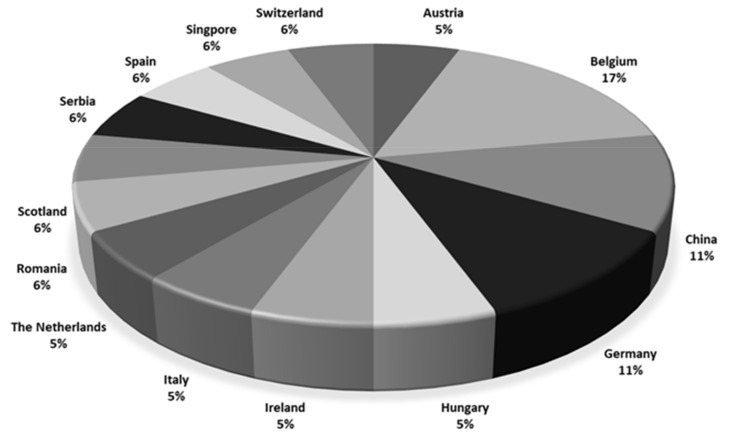
Countries of the participating laboratories (*n* = 18).

Most participants originated from Europe (Figure 1) and had up to 10 years of experience in analyzing regulated and non-regulated mycotoxins in food and feed using LC-MS/MS. Not all laboratories were accredited. Fourteen out of 18 laboratories were accredited for mycotoxin analyses, but the accreditation covered CIT in only five laboratories.

### 2.3. Participants’ Instrumental Setup

**Figure 2:**
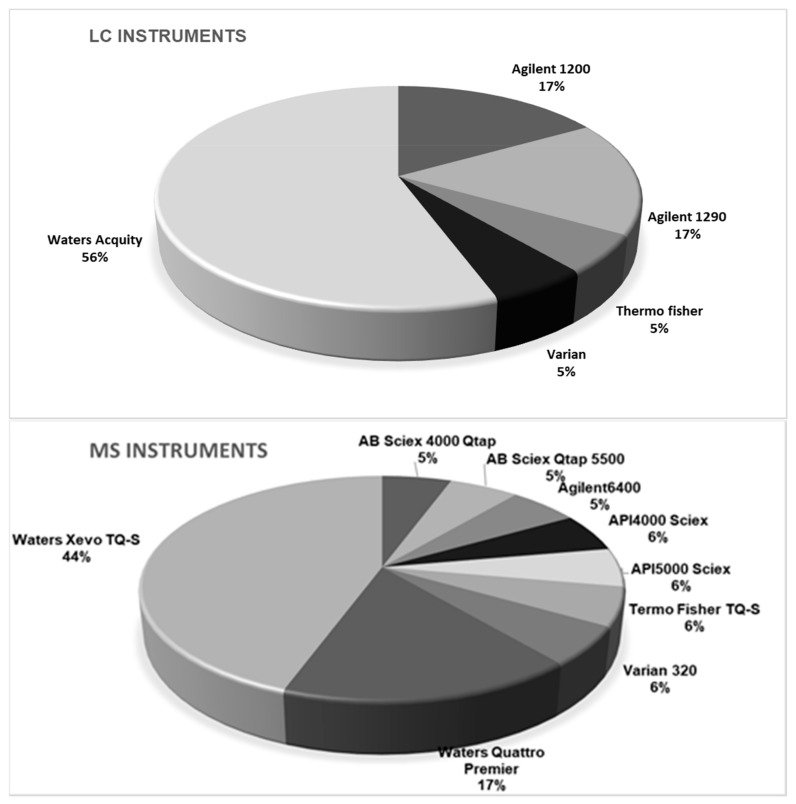

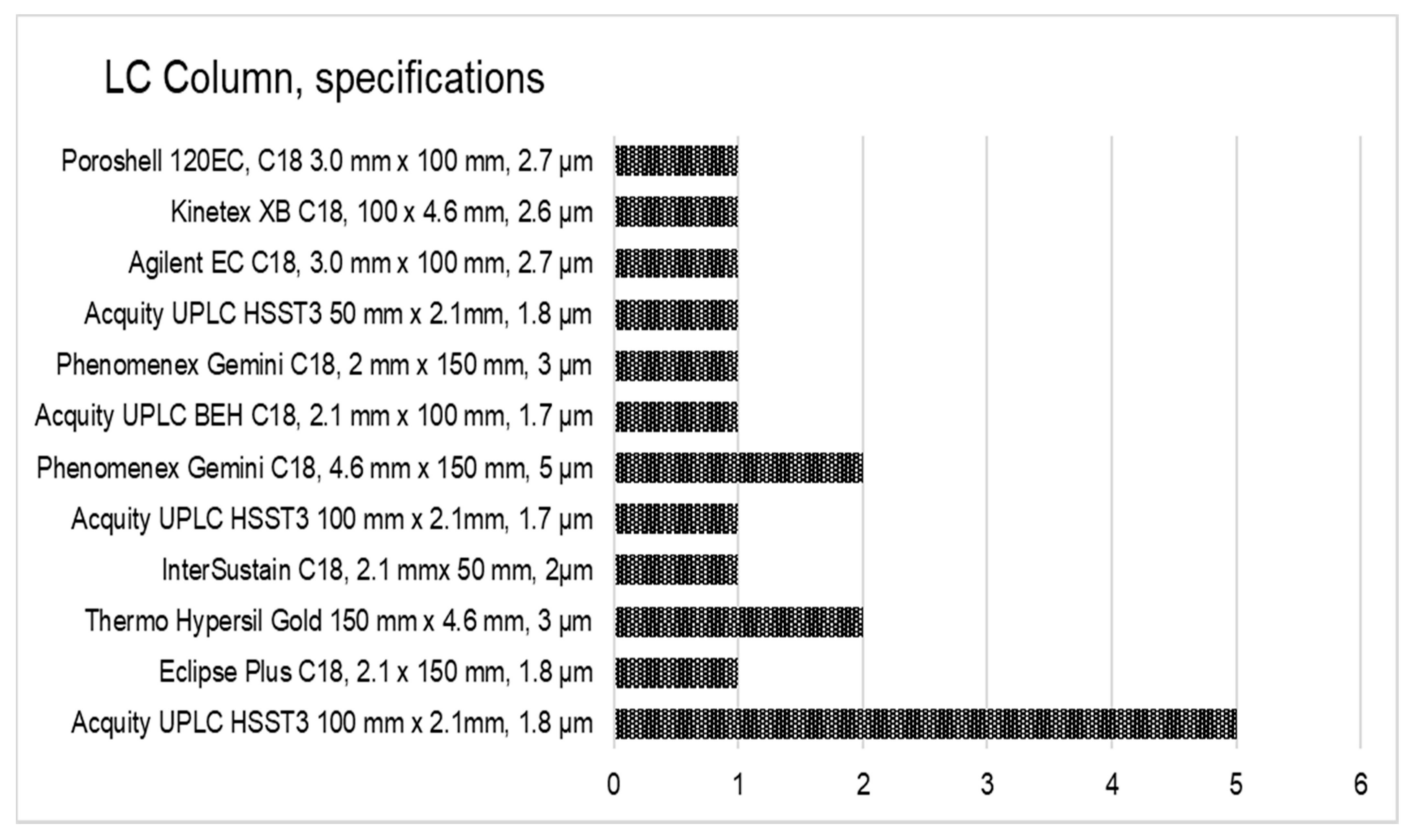
Instrumental setup used by the participating laboratories (*n* = 18).

Taking into account the diversity of instrumentation available in the laboratories, participants were free to adapt their own instrumental setup, meaning a choice of LC columns, mobile phases, and MS parameters. Conditions and MS settings of the method as achieved by participants are summarized in Figure 2. Five generations of LC instruments equipped with nine models of MS instruments and 12 different LC columns were engaged. The application of high resolution MS was accepted, but none of the participants used this technique in the method validation study. One could argue that the diversity of the instrumentation used by participants was additional proof of the applicability of the method.

While most of the participating laboratories strictly implemented the SOP under investigation, a few minor experimental modifications were reported to the organizers. The suggested mobile phases were used according to the instructions by 16 laboratories out of 18, whereas two participants modified the mobile phase by adding methanol. In contrast, the suggested gradient was used in only six laboratories, whereas 12 laboratories applied a modified gradient. Column temperature was mostly maintained at 40 °C (14 laboratories). Other column temperatures that were used by the participants were either 25 °C (one laboratory), 30 °C (two laboratories), or 50 °C (one laboratory). Injection volumes of 1 µL or 5 µL were set up by eight and seven laboratories, respectively. Other participants set up their instruments to inject either 2, 3, or 10 µL of the extracts. All instruments operated in electrospray ionization (ESI) in negative ionization mode. All participants followed the precursor and products ions as suggested in the instruction document.

### 2.4. CIT Concentrations in the Test Materials

The number of laboratories delivering results according to the instructions and the statistical evaluations of the quantitative results for CIT analysis by LC-MS/MS in the 12 materials are summarized in Table 4. Wheat flour contained a low level of CIT (< LOQ), whereas no detected level was found in RYR, GBL, FS-RYR, or FS-GBL. CIT values in contaminated samples were assigned as the consensus of the participants’ results (HorRat < 1.2) [27]. The mean CIT values assigned were 38.0 ± 3.1 µg/kg (RYR_low level_), 1913 ± 122 µg/kg (RYR_high level_), 31.1 ± 2.4 µg/kg (wheat flour), 22.1 ± 2.8 µg/kg (FS-RYR_low level_), and 30.2 ± 2.8 µg/kg (GBL). Accepted HorRat values of 1.4 and 1.7 indicated that the applied method would be able to identify the presence of CIT and estimate the degree of contamination in FS-RYR_high level_ (1867 ± 94 µg/kg) and FS-GBL (21.7 ± 3.2 µg/kg). These concentrations were in accordance with the specifications laid down in the tender (between 5–50 μg/kg for low contaminated materials and around 2000 µg/kg for the highly contaminated RYR and FS-RYR).

### 2.5. Interlaboratory Repeatability and Reproducibility Results

**Figure 3:**
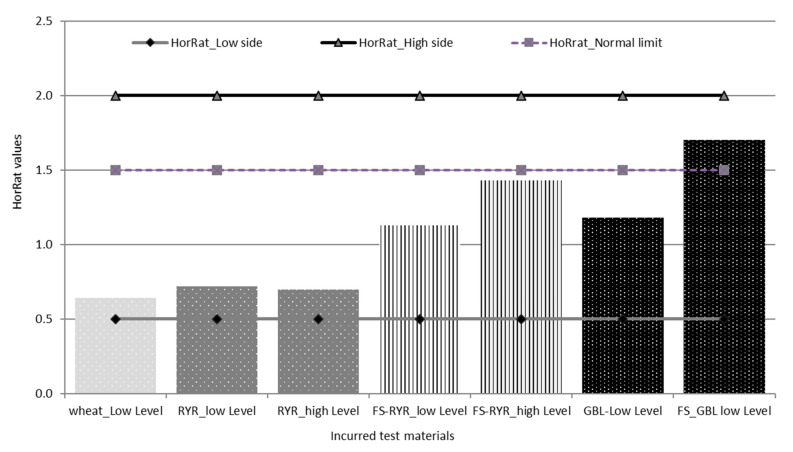
HorRat values for the tested materials.

For the contaminated materials, the precision of the method was characterized by the repeatability (RSDr) and the reproducibility (RSDR) after the removal of outliers, as recommended in ISO 5725-2 [30]. The repeatability RSDr ranged from 6.4% to 14.6% and the RSDR from 10.2 to 37.3% (Table 4). All of the repeatability results fulfilled the EC regulation 519/2014, being below the maximum acceptable RSDr (%) = 0.66 × RSDR. HorRat values were below 2.0 (Table 4, Figure 3), indicating that good reproducibility was also achieved for the validated method. Note that the empirical acceptable ranges of HorRat 0.5–2.0 requested by Horwitz and Albert [31] have met and confirmed the validity of the analytical method of the present study.

The repeatability and reproducibility limits derived for the present method validation study (Table 4) may be applicable to matrices and CIT concentration ranges as given.

However, three laboratories were not able to successfully detect CIT in the ginkgo-based materials (Samples G and L), since the ion ratios were only correct for RYR, wheat flour, and FS-RYR. One laboratory concluded that the ginkgo matrix had an effect on the signal of the ^13^C-labelled CIT internal standard and on the analyte, which consequently affected the measured ion ratios. Note that gingko products appeared to be challenging matrices to work with. The measurement and chromatography conditions were chosen in such a way that the ^13^C CIT did not produce an interfering signal quantification for the suitable mass spectrometric detection. If an adduct ion is used as the precursor ion and the loss of adduct serves as the transition for the quantifier ion, then at least two qualifier ions are monitored. Maximization of sensitivity can be achieved through optimal selection of the ionization mode, the precursor ions, and product ions, and optimization of cone voltages and collision energy.

It is worth mentioning that some organizations used HorRat as a criterion to accept the method for official purposes, as this is currently the case in the EU for aflatoxin methods for food analysis, where the only methods officially allowed are those with HorRat ≤ 2 [32].

### 2.6. Output: Future Perspective

- Within- and between-laboratory repeatability and reproducibility were acceptable, as evidenced by HorRat < 2.0. The present collaborative study demonstrated that the applied analytical method could be standardized.
- The method proved to be suitable for CIT determination in RYR, wheat flour, and FS based on either RYR or GBL at levels that could be considered by EC in the case of ML setting. Nevertheless, attention should be paid to CIT determination in *Ginkgo biloba* matrices.
- Method development and validation can be reliably improved for generating data on the occurrence of CIT in food and feed, so that exposure assessment can be improved.
- Based on the stability testing, material storage at 4 °C was recommended up to three months. Further long-term stability should be studied. Storage at higher temperatures (+24 °C) may jeopardize the stability.
- Provide information on the CIT production potential for the involved *Monascus*, *Aspergillus*, and *Penicillium* strains.
- Production and procurement of the reference materials can facilitate the toxicity studies (cell and animal experiments), so that accurate information can be generated that can be used to refine the risk assessment.

## 3. Materials and Methods

CIT-contaminated rice, wheat, and GBL were produced in-house via fungal cultivation in grains and ginkgo leaves. First, the ability to produce CIT of different strains was evaluated.

### 3.1. Fungal Screening for CIT Biosynthesis

Strains of *Monascus purpureus*, *Monascus ruber*, *Aspergillus niger*, *Aspergillus alutaceus,* and *Penicillium citrinum* from the Mycothèque de l’Université catholique de Louvain, MUCL were screened at the lab scale (three replicates of 20 g of grains per fungus) in wheat, RYR, or GBL. Next, the selected strain was inoculated and cultivated on sterilized aliquots of grains (500 g) either of wheat or RYR or on an aliquot of 1000 g of GBL, as described by Han et al. [33]. Incubation was stopped by autoclaving at 121 °C for 20 min, and samples were thereafter dried at 40–50 °C for 18 h, ground and sieved (<300 µm), homogenized, and stored at −20 °C until LC-MS/MS analyses. The results are presented in Table 5.

Based on the results, *Penicillium citrinum* (MUCL 29781) was selected to produce CIT in wheat, RYR, and GBL, as the highest concentrations of CIT were found after inoculation of these strains.

### 3.2. Collected Raw Materials

Different materials were used for the method validation study:

- Rice and wheat grains were obtained from local markets in Belgium.
- Leaves from *Ginkgo biloba* trees were collected from the Botanic Garden (Meise, Belgium).
- Capsules or tablets of food supplements (powdered RYR and GBL in bulky agents) were purchased from local drugstores in Belgium. Collected leaves were cleaned, dried, milled (as fine powder of GBL), and homogenized.
- A contaminated batch of RYR provided by the Belgian Federal Agency for Safety of Food Chain was included in the preparation of the FS-RYR materials.

The LC-MS/MS method [15] was applied for checking the CIT contents in the collected samples of wheat, RYR, GBL, FS-RYR, and FS-GBL. Briefly, a test portion (4.00 ± 0.02 g) was humidified with 10 mL of hydrochloric acid aqueous solution (water:glacial acetic acid, 99:1, *v*:*v*) and extracted with 20 mL of ethyl acetate/acetonitrile/glacial acetic acid (75:24:1, *v*:*v*:*v*) mixture for 60 min by shaking. Magnesium sulfate (6.0 g) and sodium chloride (1.5 g) were added to the extract and agitated and centrifuged in order to expel water and allow phase separation from the mixture. An aliquot of supernatant (1 mL) was collected and filtered through a PTFE syringe filter. The filtered extract (45 µL) was transferred into a glass insert, followed by the addition of the internal standard solution, ^13^C-CIT (5 µL). The mixture was homogenized and analyzed by reversed phase LC-MS/MS. Quantification was based on matching ^12^CIT/^13^C-CIT ratios and ^12^CIT concentrations.

### 3.3. Incurred Material Production

Fermented and blank (CIT < LOQ) samples of wheat and RYR were milled in a Retsch mill (ZM100 with 3.0 sieves, Haan, Germany) and homogenized to obtain particle sizes < 300 µm.

Adequate amounts of the contaminated materials were then mixed in different proportions with the corresponding blank wheat flour, RYR, or GBL powder. The test materials were carefully homogenized with EasyMIX 150 (Bellegroup, Sheen, UK) for 96 h in several steps via the cross-riffling procedure [34].

- RYR: The fermented batches of RYR with no detectable CIT level were used as RYR (blank, sample A) or mixed with the various proportion of highly CIT-contaminated RYR to produce either RYR_low level_ (sample B) or RYR_high level_ (sample C). These materials were conjointly prepared at JRC/IRMM (Geel, Belgium) for the 2015 proficiency test (PT). A surplus of these PT materials were used in this study.
- Wheat: The wheat flour with a low detectable CIT level (< LOQ) was used as sample D or mixed with highly CIT-contaminated wheat flour to prepare Wheat_low level_, sample E.
- GBL: The GBL powder with no detectable CIT level was used as blank (sample F) or mixed with the highly CIT-contaminated batch to obtain GBL_low level_ (sample G).
- FS: The tablets of FS-RYR (< LOQ) were milled and used either as blank test material (sample H) or as filling material for the preparation of sample I. This material was mixed with the highly contaminated FS-RYR to obtain FS-RYR_low level_ (sample I). Capsules of highly CIT-contaminated FS-RYR were encapsulated, homogenized, and used as FS-RYR_high level_, sample J.
- FS-GBs purchased on the market were encapsulated and used either as blank test material (sample K) or as filling material, mixed with the contaminated GBL to prepare FS-GBL_low level_ (sample L).

All of the final test materials were divided into equal portions (either 10 g or 20 g, Table 1) and in wide-neck lightproof container series 310 PVC (brown and transparent for light-sensitive media). Next, their homogeneity and stability were evaluated.

### 3.4. Homogeneity Checking

For sample B and sample C, the analyses and homogeneity testing were performed on 10 randomly selected units per material at JRC/IRMM (Geel, Belgium) [35]. For samples E, G, I, J, and L, the homogeneity was evaluated in our laboratory using 12 randomly selected units per material. Two independent CIT determinations per container were performed using the in-house validated LC-MS/MS method under repeatability conditions (same analysts, equipment and supplies, and laboratory conditions). Homogeneity was evaluated according to the criteria of ISO-13528 [26] and IUPAC [27].

### 3.5. Stability Testing

The short-term stability of the CIT in contaminated samples under storage was assessed as described by Tangni et al. [28] for ensuring whether the transport duration (three to seven days, worldwide) or the duration for laboratories to perform might not affect the CIT contents in the materials. Storage at +4 °C and +24 °C was chosen and checked against the reference temperature of −20 °C. For each storage duration (i.e., 0.25, one, and three months), two samples of each contaminated material were submitted to aging experiments under storage at −20 °C, +4 °C, and +24 °C. Two independent determinations per bottle were analyzed at random using the validated method under repeatability conditions. The mean CIT contents in samples were thus calculated for each bottle and for the two containers for each storage condition. For each temperature (e.g., +4 °C or +24 °C), the difference between the means of CIT contents in the samples stored at +4 °C (or +24 °C) against the CIT levels in reference samples stored at −20 °C was computed (|Level(+4 °C or +24 °C) − Level( −20 °C)|). In addition, the extended measurement uncertainty on the average of the two bottles was calculated under repeatability as MU_(k = 2)_ = 2 x SD_(2 averages)_/√2. The difference with the −20 °C result was considered statistically significant when it exceeded the combined MU (MU_combined_), computed using the following formula:

(2) $${\mathrm{MU}}_{\mathrm{Combined}\;(4\;{}^{\circ}C\;\mathrm{or}\;24\;{}^{\circ}C;\;-20\;{}^{\circ}C)}\;=\;\sqrt{MU_{\left(-20{}^{\circ}C\right)}{}^{2}+MU_{\left(+4{}^{\circ}C\;or+24{}^{\circ}C\right)}{}^{2}}$$

The change in CIT content expressed by ratios = |Level(+4 °C or +24 °C)– Level(−20 °C)|/MU_combined_) was considered significant when the ratio > 1.

### 3.6. Collaborative Study Management

#### 3.6.1. Recruitment of Participants

Six participating laboratories were recruited via the 2015-PT organized by IRMM at JRC, while the rest of the participants were recruited via the multi-mycotoxins PT organized by Sciensano and through specific invitation and registration of interested CEN/TC275 WG5 laboratories.

Blank samples were provided to participants to allow the laboratory to tune their LC-MS instruments for sufficient signal yield for the measurement as well as the recovery of the analyte in each material. Note that no formal pre-trial as a preliminary exercise for identifying potential bottlenecks with the method and suitable candidate laboratories for this kind of analysis was performed.

#### 3.6.2. Test Materials, Instructions, and Time Frame

Each participant received the SOP, the test materials listed in Table 1, and the standard solutions made of ^12^C-CIT (for spiking and calibration), certified ^12^C-CIT (for checking the suitability of the solution), and ^13^C-CIT (as the internal standard), separately. The amounts provided and the numbers of analyses to be performed per sample were indicated. In addition, the following documents were provided to the participants:

- an accompanying letter with instructions;
- sample receipt form;
- sample handling instructions;
- the reporting form as a protected Excel file.

The participants were also asked to fill in a questionnaire with regard to the local application of the analytical protocol and their expertise in mycotoxin analyses.

The test materials were sent to the participating laboratories at the end of January 2016, and the deadline for reporting was 31 March 2016. Five participants who encountered technical issues requested additional time for performing the analyses, therefore, the deadline for reporting was postponed until the end of April 2016.

#### 3.6.3. Data Processing and Statistical Analyses

Each participant was given a laboratory number, assigned according to the registration order. Twenty laboratories were registered. Two laboratories declared having technical problems and consequently did not submit their data. The results submitted by the 18 remaining laboratories (90%) were checked for compliance with the requested analytical procedures. Three labs did not perform the two independent CIT determinations per bottle as requested. In this group, one laboratory ran a single analysis per sample; another laboratory ran a single analysis per sample and did not analyze the blank samples, and yet another one performed a duplicate injection from the same vial. In addition, one laboratory did not submit their data for the highly contaminated RYR and FS-RYR. These four non-compliant data were excluded from the datasets because the prescribed protocol was not strictly followed.

Statistical and visual overviews of the accepted results were displayed by non-parametric box plots analysis [36] to detect graphical outliers of the analytical results. Results without outlier values were used as the reference dataset. The calculations performed and the acceptance criteria were based on the IUPAC [27].

Statistical analyses for determining the precision parameters of the CIT analyses in the different matrices were performed using the relative standard deviation for the repeatability (RSDr) as the intra-laboratory value and the relative standard deviation for reproducibility (RSDR) as the inter-laboratory value. The predicted relative reproducibility of the standard deviation PRSDR or fitness-for-purpose based standard deviations (σ) were calculated by applying the Horwitz–Thompson equation [27,29].

Note that the repeatability is the absolute difference between two single test results found on identical test material by one operator using the same apparatus within the shortest feasible time interval, and will exceed the repeatability limit r in less than 5% of the cases.

Reproducibility is mentioned to be the absolute difference between two single test results found on identical test material reported by two laboratories, exceeding the reproducibility limit R in less than 5% of the cases.

The evaluation of the collaborative study was performed using HorRat values calculated by dividing the RSDR by PRSDR (σ) [37], using the following criteria [32].

- HorRat ≤ 0.5: method reproducibility may be questionable due to the lack of study independence, unreported averaging, or consultations.
- 0.5 < HorRat ≤ 1.5: method reproducibility is as normally would be expected.
- HorRat > 1.5: method reproducibility is higher than normally expected; the study director should critically look into possible reasons for a high HorRat (e.g., were test samples sufficiently homogeneous, indefinite analyte, or property) and discuss this in the collaborative study report.
- HorRat > 2.0: method reproducibility is problematic. A high HorRat may result in rejection of a method because it may indicate unacceptable weaknesses in the method under the study.

## Figures and Tables

**Table 1:** Sample codes and provided amounts (g) of test materials.

Matrices	CIT Levels		
	Blank (< LOQ)	Low (5–50 µg/kg)	High (≈2000 µg/kg)
Food			
Red yeast rice (RYR)	Sample A, 20 g	Sample B, 20 g	Sample C, 20 g
Wheat flour (WF)	Sample D, 20 g	Sample E, 20 g	-
*Ginkgo biloba* leaves (GBL)	Sample F, 20 g	Sample G, 10 g	-
Food supplements			
Red yeast rice (FS-RYR)	Sample H, 10 g	Sample I, 10 g	Sample J, 10 g
*Ginkgo biloba* (FS-GBL)	Sample K, 10 g	Sample L, 10 g	-

LOQ = limit of quantification; -: not tested.

**Table 2:** Homogeneity of CIT-contaminated test materials.

Parameters	RYR_low level_	RYR_high level_	Wheat Flour	GBL	FS-RYR_low level_	FS-RYR_high level_	FS-GBL
Samples	B	C	E	G	I	J	L
N	10	10	12	12	12	12	12
σ (µg/kg)	3.124	175	7.43	5.76	4.42	269.22	2.27
0.3xσ	0.937	52	2.23	1.73	1.33	80.76	0.68
Sx	0.587	21	2.34	2.55	0.71	95.17	1.21
Sw	0.781	24	2.76	4.55	1.04	122.86	1.43
Ss	0.199	13	1.29	1.96	0.19	38.85	0.67
Ss < 0.3xσ	Passed	Passed	Passed	Passed	Passed	Passed	Passed
Ss^2^	0.039	166.5	1.66	3.86	0.04	1509.22	0.45
Critical value (Crit)	2.27	5736.19	17.04	26.55	4.39	27,509.85	2.93
Ss^2^ < Crit	Passed	Passed	Passed	Passed	Passed	Passed	Passed

Note: Blank samples A, D, F, H, and K are not submitted to the homogeneity testing; *n* = number of samples in the dataset; σ (%. µg/kg) = fitness for purpose-based standard deviations calculated by applying the Horwitz–Thompson function [29]. Homogeneity test: critical value = F1 × (0.3xσ) + F2 × MSw (if Ss2 < c, then the test for homogeneity has been passed). Sx = standard deviation of sample averages; Sw = within-sample standard deviation; Ss = between-sample standard deviation.

**Table 3:** Relative changes in CIT contents in samples stored at +4 °C and +24 °C compared to the samples stored at −20 °C during the study.

Samples	Codes	Relative Changes in CIT Loads in Samples					
		0.25 Months		One Month		Three Months	
		+4 °C	+24 °C	+4 °C	+24 °C	+4 °C	+24 °C
RYR_low level_	B	0.54	0.16	0.58	0.17	0.93	1.41
RYR_high level_	C	0.56	0.87	0.64	1.09	0.45	1.57
Wheat flour	E	0.45	0.26	0.20	0.21	0.55	0.42
GBL	G	0.48	0.21	0.06	0.13	1.12	1.41
FS-RYR_low level_	I	0.64	1.17	0.57	1.12	1.02	0.93
FS-RYR_high level_	J	0.49	0.34	0.63	1.24	0.29	0.11
FS-GBL	L	0.17	0.19	0.44	0.01	0.65	0.29

**Table 4:** Statistical results of the interlaboratory trial for CIT analysis in the 12 test materials.

	Red Yeast Rice			Wheat		*Ginkgo Biloba* Leaves (GBL)		Food Supplement—RYR (FS-RYR)			Food Supplement—GBL (FS-GBL)	
	A	B	C	D	E	F	G	H	I	J	K	L
Statistical parameters	Blank	RYR_low level_	RYR_high level_	Blank	Wheat_low level_	Blank	GBL_low level_	Blank	FS-RYR_low level_	FS-RYR_high level_	Blank	FS-GBL_low level_
Number of laboratories delivering results	17	18	17	17	18	17	18	17	17	17	17	15
Number of laboratories with results per requested	14	14	14	14	14	14	14	14	14	14	14	12 **
Number of outliers	0	1	1	0	1	3	3	0	1	0	1	2
Number of accepted results	14	13	13	14	13	11	11	14	13	14	13	10
Mean value (µg/kg)	0.1	38.0	1913.3	1.4	31.1	0.1	30.2	0.0	22.1	1866.5	0.0	21.7
Median value (µg/kg)	0.0	39.4	1956.0	1.6	31.1	0.0	29.2	0.0	21.3	1973.9	0.0	22.6
Repeatability SD S_r_ (µg/kg)	-	3.1	122.0	-	2.4	-	2.8	-	2.8	93.7	-	3.2
Repeatability RSD_r_ (%)	-	8.1%	6.4%	-	7.6%	-	9.2%	-	12.5%	5.0%	-	14.6%
Repeatability limit r (µg/kg)	-	8.7	341.5	-	6.6	-	7.8	-	7.7	262.3	-	8.9
Reproducibility SD S_R_ (µg/kg)	-	6.0	194.2	-	4.4	-	7.5	-	5.4	387.5	-	8.1
Reproducibility RSD_R_ (%)	-	15.8%	10.2%	-	14.1%	-	24.9%	-	24.9%	20.8%	-	37.3%
Reproducibility limit R (µg/kg)	-	16.8	543.8	-	12.3	-	21.0	-	15.4	1085.0	-	22.7
Mean recovery (%)	-	80.8%	-	-	89.8%	-	74.9%	-	78.8%	-	-	69.2%
Horwitz−Thompson value (µg/kg)	-	8.4	277.6	-	6.9	-	6.6	-	4.9	271.9	-	4.8
Horwitz−Thompson value (%)	-	22.0%	14.5%	-	22.0%	-	22.0%	-	22.0%	14.6%	-	22.0%
HorRat values *	-	0.72	0.70	-	0.64	-	1.18	-	1.13	1.43	-	1.70

* HorRat values are computed with the accepted results; ** one laboratory reported that 13C was not detected, and one other laboratory reported ion suppression for this matrix. Moreover, two outliers were identified, and corresponded to the results obtained with a bad ion ratio.

**Table 5:** Fungal screening for production of CIT and OTA in wheat, rice grains, and GB leaves.

Strains (MUCL Nomenclature)		CIT (µg/kg)			OTA (µg/kg)		
MUCL		Wheat	Rice	GBL	Wheat	Rice	GBL
*Monascus purpureus*	51640	ND	ND	ND	Trace	trace	8
	53806	434	242	ND	8	34	6
	53807	98	48	ND	Trace	7	5
	53808	17	23	ND	77	6	4
*Monascus ruber*	53809	ND	ND	ND	Trace	trace	9
*Penicillium* *citrinum*	29781	3934	1419	1183	ND	9	2
	31475	1952	589	277	81	3	3
*Aspergillus* *alutaceus*	21683	3	Trace	ND	3	5168	4
	39539	9	ND	ND	25,869	47,046	299
	44480	ND	ND	ND	54	24	7
	44481	ND	ND	ND	23	7	6
*Aspergillus* *niger*	13608	ND	ND	ND	11	7	3
	15973	ND	ND	ND	23	30	38
	18911	ND	ND	ND	29	15	2
	35442	ND	ND	ND	9	ND	4

CIT = Citrinin; OTA = Ochratoxin A; Limit of detection, LOD = 0.8 µg/kg and LOQ = 2.5 µg/kg (Not detected, ND: CIT content < LOD; Trace: LOD < CIT contents < LOQ); MUCL = Mycothèque de l’Université catholique de Louvain.
